# COVID-19 disease severity in US Veterans infected during Omicron and Delta variant predominant periods

**DOI:** 10.1038/s41467-022-31402-4

**Published:** 2022-06-25

**Authors:** Florian B. Mayr, Victor B. Talisa, Alexander D. Castro, Obaid S. Shaikh, Saad B. Omer, Adeel A. Butt

**Affiliations:** 1 VA Pittsburgh Healthcare System, Pittsburgh, PA USA; 2 CRISMA Center, Department of Critical Care Medicine, University of Pittsburgh, Pittsburgh, PA USA; 3 University of Pittsburgh School of Medicine, Pittsburgh, PA USA; 4 Department of Medicine, Division of Gastroenterology, University of Pittsburgh, Pittsburgh, PA USA; 5 Institute for Global Health, Yale University, New Haven, CT USA; 6 Department of Medicine, Weill Cornell Medicine, New York, NY USA; 7 Department of Population Health Sciences, Weill Cornell Medicine, New York, NY USA; 8 Department of Medicine, Weill Cornell Medicine, Doha, Qatar; 9 Department of Population Health Sciences, Weill Cornell Medicine, Doha, Qatar

**Keywords:** Epidemiology, Epidemiology, SARS-CoV-2, Risk factors

## Abstract

The SARS-CoV-2 Omicron variant is thought to cause less severe disease among the general population, but disease severity among at-risk populations is unknown. We performed a retrospective analysis using a matched cohort of United States veterans to compare the disease severity of subjects infected during Omicron and Delta predominant periods within 14 days of initial diagnosis. We identified 22,841 matched pairs for both periods. During the Omicron period, 20,681 (90.5%) veterans had mild, 1308 (5.7%) moderate, and 852 (3.7%) severe disease. During the Delta predominant period, 19,356 (84.7%) had mild, 1467 (6.4%) moderate, and 2018 (8.8%) severe disease. Moderate or severe disease was less likely during the Omicron period and more common among older subjects and those with more comorbidities. Here we show that infection with the Omicron variant is associated with less severe disease than the Delta variant in a high-risk older veteran population, and vaccinations provide protection against severe or critical disease.

## Abstract (web summary)

Infection with the SARS-CoV-2 Omicron variant appears to cause milder disease in the general population than infection with Delta. Here the authors use data from the US Department of Veterans Affairs and demonstrate that this also applies to infection in an at-risk population of older age and with more co-morbidities.

## Introduction

First reported from South Africa in November 2021, the Omicron variant rapidly became the predominant SARS-CoV-2 strain globally in a short time^1^. While the Omicron variant is more infectious than previous variants, several reports from South Africa and European countries suggest that it is associated with milder disease^2–6^. Current vaccines are less effective against the Omicron variant than against previous variants^7^. Vaccine effectiveness against symptomatic infection and severe disease is higher in persons who were boosted after completing a primary vaccination series than those who were not^8–10^.

Whether SARS-CoV-2 disease severity in high-risk populations infected with the Omicron variant differs from prior variants such as Delta remains incompletely characterized. Specific risk factors associated with a higher risk for severe disease in persons infected with the Omicron variant are also poorly understood. Compared to the general population, veterans in the United States who receive their healthcare through the Veterans Health Administration (VHA) are at a higher risk of infection and severe disease with SARS-CoV-2 due to their older age and higher burden of preexisting comorbidities^11,12^.

In this work, we compare characteristics and clinical outcomes of veterans with polymerase chain reaction (PCR) confirmed SARS-CoV infections during Omicron vs. Delta predominant periods. We show that infection with the Omicron variant is associated with significantly lower disease severity in a high-risk national population as measured by hospitalization rates and need for intensive care unit admission, organ support measures, or death. Vaccination, in particular an additional booster dose, continues to offer strong protection against severe or critical disease from the Omicron variant.

## Results

### Patient characteristics

**Fig. 1:**
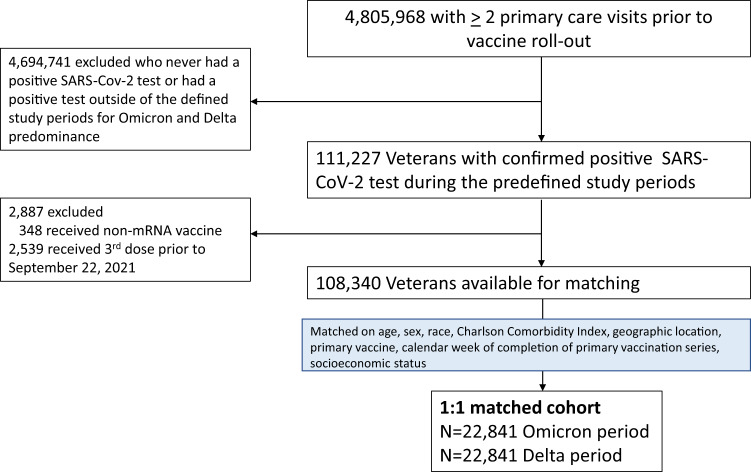
Overview of study cohort derivation. We constructed a 1:1 matched cohort by matching veterans infected during the Omicron variant period with veterans infected during the Delta variant period using random coarsened exact matching. Individuals were matched on age, sex, race, vaccination status at the time of infection, second vaccine dose administration date, Charlson Comorbidity Index, area deprivation score (as a marker of socioeconomic status), and VA medical center to account for local differences in SARS-CoV-2 transmission, testing, and hospital admission practices.

Our matched analysis dataset consisted of 22,841 veterans infected during the Omicron period and 22,841 matched veterans infected during the Delta period. (Fig. 1) The median age (IQR) was 62.0 years (49.0, 72.0), 91.9% were men, and 82.4% were White. The majority of veterans in our cohort were multi-morbid, and more than 75% (34,492/45,682) had two or more preexisting chronic health conditions. The median Charlson Comorbidity Index score was 3 (IQR 2, 4).

Among both groups, 7393 (32.4%) had received two doses of an mRNA vaccine, and 990 (4.3%) had received an additional booster dose (Table 1). Infection was diagnosed ≥14 days after the booster dose in 910 (4.0%) of those infected during the Omicron period and 403 (1.8%) of those infected during the Delta period.

**Table 1:** Baseline characteristics of propensity score-matched veterans infected during Omicron and Delta predominant periods.

Characteristic	Omicron predominant infection period *N* = 22,841	Delta predominant infection period *N* = 22,841
Age		
Median (IQR)	62.0 (49.0, 72.0)	62.0 (49.0, 72.0)
18–39 years	3370 (14.8)	3370 (14.8)
40–49 years	2595 (11.4)	2595 (11.4)
50–59 years	4161 (18.2)	4161 (18.2)
60–69 years	4,904 (21.5)	4904 (21.5)
70–79 years	6453 (28.3)	6453 (28.3)
80–89 years	1176 (5.1)	1176 (5.1)
≥ 90 years	182 (0.8)	182 (0.8)
Sex		
Male	21,000 (91.9)	21,000 (91.9)
Female	1841 (8.1)	1841 (8.1)
^a^Race		
White	18,811 (82.4)	18,811 (82.4)
Black	2638 (11.5)	2638 (11.5)
Other or missing	1392 (6.1)	1392 (6.1)
Body Mass Index (median, IQR)	30.0 (26.5, 34.1)	30.2 (26.6, 34.4)
Charlson Comorbidity Index (median, IQR)	3 (2, 4)	3 (2, 4)
Comorbidities		
Hypertension	13,369 (58.5)	13,538 (59.3)
Diabetes	6447 (28.2)	6863 (30.0)
Chronic obstructive pulmonary disease	5002 (21.9)	5033 (22.0)
Chronic kidney disease	2449 (10.7)	2575 (11.3)
Congestive heart failure	1998 (8.7)	2094 (9.2)
Malignancy	2062 (9.0)	1974 (8.6)
Vaccination status at the time of infection		
Not vaccinated at time of infection	12,977 (56.8)	12,977 (56.8))
Received at least one vaccine dose at the time of infection	9864 (43.2)	9864 (43.2)
Vaccinated with only 1 dose at time of infection	1481 (6.5))	1481 (6.5)
Vaccinated with 2nd dose < 3 months prior to infection	44 (0.2)	85 (0.4)
Vaccinated with 2nd dose ≥ 3 months prior to infection	7349 (32.2)	7308 (32.0)
Vaccinated with 3rd dose prior to infection	990 (4.3)	990 (4.3)
Infection status in relation to vaccination		
Infection before or up to 14 days of 2nd dose	1488 (6.5)	1509 (6.6)
Infection post 14 days of 2nd dose up to 14 days post 3rd dose	7466 (32.7)	7952 (34.8)
Infection ≥ 14 days after 3rd dose	910 (4.0%)	403 (1.8%)

^a^Detailed racial categories for both groups are provided in Supplementary Table 6.

### Disease severity

Among those infected during the Omicron period, 20,681 (90.5%) had mild disease, whereas 1308 (5.7%) fulfilled moderate disease criteria, and 852 (3.7%) met severe/critical disease criteria. Among those infected during the Delta period, 19,356 (84.7%) had mild disease, 1467 (6.4%) fulfilled moderate disease, and 2018 (8.8%) severe/critical criteria, respectively. Overall, a significantly lower proportion of veterans met moderate or severe/critical disease criteria during the Omicron period than Delta period (9.5% vs. 15.3%; *p* < 0.001; Table 2).

**Table 2:** Summary of disease outcomes of the two SARS-CoV-2 variant groups.

	Omicron variant *N* = 22,841	Delta variant *N* = 22,841	*P*-value
	*N* (%)	*N* (%)	
Outcome-disease status			
Mild	20,681 (90.5)	19,356 (84.7)	2.2 × 10^−16^
Moderate	1308 (5.7)	1467 (6.4)	
Severe/critical	852 (3.7)	2018 (8.8)	
Moderate or severe/critical outcome	2160 (9.5)	3485 (15.3)	2.2 × 10^−16^

Moderate disease: Hospitalized but no intensive care unit admission; Severe/critical disease: intensive care unit admission or death with 28 days of positive test date

Comparisons between groups were performed using Pearson’s X^2^ tests. A two-sided *P*-value of < 0.05 was considered to be statistically significant. Adjustments for multiple comparisons were not made.

Of the 2160 moderate or severe/critical infections during the Omicron period, 48 (2.2%) occurred in those who had received a booster dose ≥14 days prior. Of the 3485 moderate or severe/critical infections during the Delta period, 58 (1.7%) occurred among individuals who had received a booster mRNA vaccine at least 14 days before their infection. (*P* < 0.001; Table 3)

**Table 3:** Summary of disease outcomes of the two SARS-CoV-2 variant groups stratified by vaccination status.

	Infection among veterans without booster (≥14 days of 2nd dose and <14 days post 3rd dose)			Infection among veterans with booster (≥ 14 days of 3rd dose)		
	Omicron *N* = 7,466	Delta *N* = 7,952	*P*-value	Omicron *N* = 910	Delta *N* = 403	*P*-value
	*N* (%)	*N* (%)		*N* (%)	*N* (%)	
Outcome-disease status						
Mild	6760 (90.5)	6826 (85.8)	2.2 × 10^−16^	849 (93.3)	345 (85.6)	1.233 × 10 ^−5^
Moderate	451 (6.0)	572 (7.2)		44 (4.8)	34 (8.4)	
Severe-critical	255 (3.4)	554 (7.0)		17 (1.9)	24 (6.0)	
Moderate or severe	706 (9.4)	1126 (14.2)	2.2 × 10^−16^	48 (5.7)	58 (14.4)	1.731 × 10^-4^

Moderate disease: Hospitalized but no intensive care unit admission; Severe/critical disease: intensive care unit admission or death with 28 days of positive test date

Comparisons between groups were performed using Pearson’s X^2^ tests. A two-sided *P*-value of < 0.05 was considered to be statistically significant. Adjustments for multiple comparisons were not made.

In the multivariable logistic regression model, infection during the Omicron period was associated with lower odds of moderate or severe disease (unadjusted odds ratio: 0.58, 95% CI 0.55 to 0.62, *P* < 2 × 10^−16^; adjusted odds ratio (aOR): 0.56; 95% CI 0.53–0.59, *P* < 2 × 10^−16^).

### Additional analyses

Baseline characteristics of the entire cohort before matching (72,492 in the Omicron period and 35,848 in the Delta variant period) are presented in Supplementary Table 2. Veterans with confirmed COVID-19 disease during the Omicron period were younger, and a higher proportion was female and non-White. Fewer veterans were unvaccinated during the Omicron period (33.5% vs. 48.8%), and a significantly higher proportion had received a booster vaccine (19.4% Omicron vs. 4.1% Delta).

We calculated the odds of the disease severity stratified by the predominant variant. Vaccination was associated with significant protection against moderate or severe/critical disease. The unadjusted OR was 0.99 (95% CI 0.89–1.09, *P* = 0.786), and the aOR was 0.51 (95% CI 0.46–0.57, *P* < 2 × 10^−16^) for Omicron variant infection among vaccinated with 2nd dose ≥ 3 months before infection. The unadjusted OR was 0.68 (95% CI 0.51–0.87) and the aOR was 0.26 (95% CI 0.20–0.34, *P* < 2 × 10^−16^) for recipients of a booster dose ≥ 14 days before infection. The corresponding unadjusted and adjusted ORs for the Delta variant period were 0.85 (95% CI 0.78–0.92, *P* = 5.47 × 10^−5^ and 0.47 (95% CI 0.43–0.51, *P* < 2 × 10^−16^) for those vaccinated with a 2nd dose ≥ 3 months before infection, respectively. Accordingly, for recipients of a booster dose ≥ 14 days before infection, the unadjusted OR for severe/critical disease was 0.82 (95% CI 0.60–1.09, *P* = 0.177), and the aOR was 0.34 (95% CI 0.25–0.46, *P* = 3.46 × 10^−12^).

The proportion of veterans requiring organ support measures during Omicron and Delta variant periods are summarized in Supplementary Table 3. A significantly lower proportion of individuals in the Omicron variant period required supplemental low flow oxygen (36.3% vs. 63.4%), high flow oxygen (8.8% vs. 25.9%), and mechanical ventilation (6.5% vs. 10.0%). (*P* < 0.001 for all comparisons). The need for incident renal replacement therapy and vasopressor support did not differ between Omicron and Delta predominant periods.

### Sensitivity Analyses

We recalculated the proportion of persons in each disease severity category by applying a more stringent definition of variant predominance, limiting time periods when each variant constituted >98% of all reported variants (October 1 to December 4, 2021, for the Delta variant and January 2 to January 15, 2022, for the Omicron variant). Results from our sensitivity analysis of 19,874 matched pairs were similar to our primary results (Supplementary Table 4).

To exclude a potential confounding effect of prior treatment with monoclonal antibodies or nitravelmir/ritonavir (paxlovid) on disease severity, we performed sensitivity analyses and excluded 3861 patients who had received these treatments after a positive SARS-CoV-2 PCR test. Disease severity estimates of 21,231 matched pairs were similar to our primary analysis (Supplementary Table 5).

Similarly, we performed additional analyses to exclude confounding due to different hospital bed capacities during Omicron and Delta periods. Assessing bed capacity in this context is challenging because the number of authorized beds does not necessarily equate to the number of staffed beds, i.e., beds with nursing and other staff available to accommodate patients. We had information about acute medical and surgical care beds in operation for 107 of 129 VA facilities included in our dataset before matching. The average number of daily admissions for these facilities was well below operating bed capacity and thus unlikely to confound our estimates. The median number of admissions per day during the 24-day Omicron period was 1.62 (IQR 0.87, 2.67). In contrast, the median number of daily admissions during the 72-day Delta period was 0.49, IQR: 0.29, 0.76), resulting in a median daily admission ratio (Omicron: Delta) of 3.25 (IQR 1.74, 5.25). We performed sensitivity analysis on the matched data and calculated disease severity estimates for facilities with median daily Omicron: Delta admission ratios below and above the 50% percentile (<3.25 vs. ≥3.25). Results were similar in both strata, suggesting that acute care bed capacity did not affect our severity estimates substantially.

## Discussion

Early reports suggest that persons infected with the SARS-CoV-2 Omicron variant may be at a lower risk for adverse clinical disease trajectories than earlier variants of concern^3–6^. However, these findings may not be generalizable to US veterans due to significantly younger study populations, lower comorbidity burden, and different uptake of vaccinations, particularly booster vaccines. We add to these studies by providing results from a national high-risk cohort of older veterans in the US and describe clinical outcomes during Omicron and Delta predominant periods.

We found that individuals infected with the Omicron variant were less than half as likely to experience severe/critical disease in the 14-day period after diagnosis compared with those infected with the Delta variant (crude proportions 3.7% vs. 8.8%; aOR 0.38, 95% CI 0.35–0.42). Persons infected with the Omicron variant were also less likely to require hospital admission than those infected with the Delta variant (aOR 0.89, CI: 0.82–0.97). Those infected during the Omicron period were also less likely to require respiratory organ support (supplemental oxygen and mechanical ventilatory support). Our results remained unchanged in several sensitivity analyses. Our data confirm earlier reports that infection with the Omicron variant is associated with less severe disease, even in at-risk older persons^5,6^. These data are important in planning service delivery for the ongoing Omicron wave and may also help plan infection prevention and control measures. The reason for lesser severity with Omicron variant infection is not yet known. Many novel mutations may have attenuated its virulence, and in vitro studies suggest that the Omicron variant has lower replication and fusion efficiency than the Delta variant, which may partly explain these findings^13^.

Most moderate or severe/critical disease events occurred in individuals who had not received a booster vaccination. While this was true for both Omicron and Delta variant infections, the protection from the booster dose was more pronounced in the Omicron variant period. This finding suggests that a booster dose is highly protective against moderate and severe/critical disease from Omicron variant infection. Older age and a higher comorbidity burden are well-known factors associated with adverse outcomes and more severe disease in patients with COVID-19 infection^14^.

Our study has several strengths, including a large sample size of US residents across 50 states, data collection from multiple sources in one of the largest integrated healthcare systems, and adjustment for important clinical confounding variables. We also performed sensitivity analyses to exclude a confounding effect on severity estimates due to differences in bed capacity and treatment with monoclonal antibodies and other antiviral therapies. Our study also has certain limitations. First, it is a retrospective study and susceptible to residual confounding. To mitigate potential bias from an imbalance in baseline risk factors, we conducted matching of the two study groups. Second, we did not have individual-level variant sequencing data available. We, therefore, used weighted variant proportions provided by the Centers for Disease Control and Prevention to identify Delta and Omicron predominant periods (>99% for Delta and >90% for Omicron infections, respectively), which could have resulted in misclassification in individual instances. However, our results remained essentially unchanged in sensitivity analyses using a more conservative threshold to define variance predominance >98% for both variants. Third, our study cohort represents a male-dominant older cohort of United States veterans with 82% White and 11% Black make-up from all geographic areas of the US. Therefore, our results are likely not generalizable to women, younger populations, and middle/low-income countries. We restricted our analyses to veterans who had been vaccinated with mRNA vaccines. Hence, caution is necessary when extrapolating our results to settings where additional or other vaccines are available.

In summary, infection with the Omicron variant is associated with significantly lower disease severity in a high-risk national population as measured by hospitalization rates and need for intensive care unit admission or death. Vaccination, in particular an additional booster dose, continues to offer strong protection against severe or critical disease from the Omicron variant.

## Methods

### Study setting

In response to the SARS-CoV-2 pandemic, the Veterans Health Administration (VHA) rapidly created a national COVID-19 Shared Data Resource. This resource contains information on all veterans with a laboratory-confirmed diagnosis of SARS-CoV-2 infection and vaccine receipt within the VA. Information of veterans tested or vaccinated outside VA is captured by patient self-report (presentation of a vaccination card) or through insurance claims data. The VA COVID-19 Shared Data Resource is updated regularly and contains extensive demographic and clinical information, including self-reported race and ethnicity (collected as two separate questions), receipt of a vaccine with type and date of each dose, laboratory data, vital signs, and clinical outcomes information derived from multiple validated sources^14–16^.

### Variant identification

Based on nationally representative sequencing data reported by the US Centers for Disease Control and Prevention, we identified periods during which the Delta and the Omicron were the predominant variants circulating in the US^17^. Between October 1 and December 11, 2021, the Delta variant constituted >98% of the identified strains nationally, and veterans with a confirmed SARS-CoV-2 infection during that time were considered infected with the Delta variant. Between December 26, 2021, and January 15, 2022, 88.9–99.6% of infections were due to the Omicron variant, and those diagnosed during this period were considered to be infected with the Omicron variant. (Supplementary Table 1)

### Study population

All veterans with a confirmed SARS-CoV-2 infection based on a positive RT-PCR on a nasopharyngeal swab between October 1, 2021 and January 15, 2022 were eligible for inclusion. We retained those with at least two primary-care appointments in the preceding 18 months of vaccine rollout (because these individuals are more likely to receive care, including vaccinations, through the VA). We excluded veterans who had evidence of prior SARS-CoV-2 infection and veterans who had received non-mRNA vaccines since the mRNA vaccines were the predominant vaccine types administered to the VA population. Within this group, we matched each person in the Omicron variant period to a person in the Delta variant period using random coarsened exact matching. Individuals were matched on age, sex, race, vaccination status at the time of infection, second vaccine dose administration date, Charlson Comorbidity Index, area deprivation score (as a marker of socioeconomic status), and VA medical center to account for local differences in SARS-CoV-2 transmission, testing, and hospital admission practices.

### Outcomes

The primary outcome of interest was the severity of COVID-19 disease in persons infected with the Omicron variant compared with those infected with the Delta variant within 14 days of their index positive SARS-CoV-2 test. We classified disease severity into three categories: mild disease, defined as PCR confirmed infection without documented hospitalization; moderate disease, defined as persons with confirmed infection requiring hospitalization but no admission to an intensive care unit (ICU); and severe/critical disease, defined as the need for ICU admission, or death within 28 days of SARS-COV-2 positive test results^18^.

### Statistical analyses

We conservatively estimated that 15% of veterans with Delta infection would experience moderate disease to determine the sample size^19,20^. Considering a 25% reduction in this outcome to be clinically relevant, we estimated that a minimum sample size of 2544 (1272 in each group) would detect the difference at an alpha level of 0.05 with a power of 80%.

Proportions of veterans with mild, moderate, or severe/critical disease among those infected during the Omicron and the Delta variant periods were calculated and compared overall and stratified by vaccination status. 95% confidence intervals (CIs) were calculated to express the uncertainty around the point estimate. We used logistic regression models accounting for matching, gender, age, number of chronic health conditions, vaccination status, week of 2nd vaccination, socioeconomic status, and VA medical center to calculate adjusted odds ratios (aORs) and 95% CIs for factors associated with primary and secondary outcomes. We selected variables based on prior associations with disease severity or outcomes. We did not adjust for multiple comparisons. All analyses were performed using R 4.0.5, and a two-sided *p*-value of ≤ 0.05 was considered statistically significant.

### Reporting summary

Further information on research design is available in the Nature Research Reporting Summary linked to this article.

## Supplementary information

Supplementary Information

Reporting Summary

## Data Availability

The data that support the findings of this study are available from the VA. VA data are made freely available to researchers behind the VA firewall with an approved VA study protocol. More information is available at https://www.virec.research.va.gov or the VA Information Resource Center (VIReC) at VIReC@va.gov.

## Code availability

The R programming code used for data analysis is available at https://github.com/mayrfb/Omicron_Delta_Severity.
