# Supplementary material for: COVID-19 disease severity in US Veterans infected during Omicron and Delta variant predominant periods
Source: Nat Commun. 2022 Jun 25;13:3647. doi: 10.1038/s41467-022-31402-4 (PMC9233663; doi:10.1038/s41467-022-31402-4)
Supplement: Supplementary file 1 — Supplementary Information [file 41467_2022_31402_MOESM1_ESM.pdf]

Supplementary Information

COVID-19 Disease Severity in US Veterans infected During Omicron and Delta variant predominant periods

Supplementary tables

Supplementary Table 1. Proportion of circulating Omicron and Delta variants in the United States over time

Week Ending	Omicron % (95% CI)	Delta % (95% CI)
10/16/2021	0	99.2 (98.8-99.5)
10/23/2021	0	99.0 (98.6-99.4)
10/30/2021	0	99.3 (98.9-99.5)
11/06/2021	0	98.7 (98.2-99.2)
11/13/2021	0	98.6 (98.0-99.0)
11/20/2021	0	98.4 (97.8-98.9)
11/27/2021	0.1 (0.0-0.2)	99.1 (98.6-99.4)
12/04/2021	0.6 (0.4-0.9)	99.2 (98.8-99.5)
12/11/2021	7.4 (4.9-10.6)	92.4 (89.2-94.9)
12/18/2021	37.8 (32.8-43.0)	61.8 (56.6-66.8)
12/25/2021	74.1 (69.3-78.5)	25.2 (21.0-29.9)
01/01/2022	88.9 (86.9-90.6)	10.0 (8.3-11.8)
01/08/2022	96.2 (94.9-97.3)	3.8 (2.7-5.1)
01/15/2022	99.4 (99.2-99.6)	0.6 (0.4-0.8)
01/22/2022	99.9 (99.8-99.9)	0.1(0.1-0.2)

(Source: http://www.covid.cdc.gov, accessed Feb 1, 2022)

Supplementary Table 2. Baseline characteristics of all veterans with a positive SARS-CoV-2-PCR test during periods of Omicron and Delta variant predominance

	Omicron N=72,492	Delta N=35,848
Age		
Median (IQR), years	58.0 (44.0, 70.0)	64.0 (50.0, 73.0)
18-39 years	13,074 (18.0)	4,555 (12.7)
40-49 years	10,787 (14.9)	3,957 (11.0)
50-59 years	14,979 (20.7)	6,130 (17.1)
60-69 years	15,109 (20.8)	7,443 (20.8)
70-79 years	14,546 (20.1)	10,262 (28.6)
80-89 years	3,145 (4.3)	2,785 (7.8)
≥ 90 years	852 (1.2)	716 (2.0)
Sex		
Female	11,225 (15.5)	3,822 (10.7)
Male	61,267 (84.5)	32,026 (89.3)
Race		
White	44,299 (61.1)	28,517 (79.5)
Black	20,057 (27.7)	4,001 (11.2)
Other / unkown	8,136 (11.2)	3,330 (9.3)
Comorbidities		
Hypertension	38,638 (53.3)	20,995 (58.6)
Diabetes	18,172 (25.1)	10,344 (28.9)
Chronic obstructive lung disease	13,428 (18.5)	7,629 (21.3)
Chronic kidney disease	6,776 (9.3)	4,049 (11.3)
Congestive heart failure	5,123 (7.1)	3,156 (8.8)
Malignancy	5,545 (7.7)	3,158 (8.8)
Comorbidities		
Median Charlson Comorbidity Index, IQR	3 (1, 4)	3 (1, 4)
None	7,235 (10.0)	3,138 (8.8)
1	13,693 (18.9)	6,243 (17.4)
2 or more	51,564 (71.1)	26,467 (73.8)
Vaccination status at time of infection		
Not vaccinated at time of infection	24,317 (33.5)	17,481 (48.8)
Vaccinated with only 1 dose at time of infection	5,440 (7.5)	2,712 (7.6)
Vaccinated with 2nd dose <3 months prior to infection	1,493 (2.1)	357 (1.0)
Vaccinated with 2nd dose >= 3 months prior to infection	27,150 (37.5)	13,813 (38.5)
Vaccinated with 3rd dose prior to infection	14,092 (19.4)	1,485 (4.1)
Infection status in relation to vaccination		
Infection before or < 14 days of 2nd dose	5,644 (7.8)	2,818 (7.9)
Infection ≥14 days of 2nd dose and <14 days post 3rd dose	29,955 (41.3)	14,953 (41.7)
Infection ≥14 days after 3rd dose	12,576 (17.3)	596 (1.7)

Supplementary Table 3. Proportion of Veterans with moderate or severe/critical disease requiring organ support during Omicron and Delta variant predominant periods.

	Omicron (N=2,160)	Delta (N=3,485)	P value
Low flow oxygen	785 (36.3)	2,208 (63.4)	2.2×10^{-16}
High flow oxygen	189 (8.8)	901 (25.9)	2.2×10^{-16}
Mechanical ventilation	140 (6.5)	347 (10.0)	7.8×10^{-6}
Extracorporeal membrane oxygenation	0 (0)	0 (0)	n.a.
Renal Replacement Therapy	74 (3.4)	100 (2.9)	0.273
Vasopressors	168 (7.8)	304 (8.7)	0.231

Comparisons between groups were performed using Pearson's X^2 tests with Yates' continuity correction. A two-sided P-value of < 0.05 was considered to be statistically significant. Adjustments for multiple comparisons were not made.

Supplementary Table 4. Summary of disease outcomes of the two SARS-CoV-2 variant groups using alternative time periods for defining Omicron and Delta predominance (>95% of circulating variants).

	Omicron variant N=19,874	Delta variant N=19,874	P-value
	N (%)	N (%)	
Outcome-disease status			$2.2x10^{-16}$
Mild	16,931 (85.2)	18,167 (91.4)	
Moderate	1,090 (5.5)	1,248 (6.3)	
Severe/critical	617 (3.1)	1,695 (8.5)	
Moderate or severe/critical outcome	1,707 (8.6)	2,943 (14.8)	

Delta period was defined as time period between 10/1/2021 and 12/4/2021; Omicron period was defined as time period between 1/2/2022 and 1/15/2022.

Moderate disease: Hospitalized but no intensive care unit admission; Severe/critical disease: intensive care unit admission or death within 28 days of positive test.

Comparison between groups was performed using Pearson's X^2 test. A two-sided P-value of < 0.05 was considered to be statistically significant. Adjustments for multiple comparisons were not made.

Supplementary Table 5. Disease severity estimates after excluding veterans treated with monoclonal antibodies and/or nitravelmir/ritonavir (paxlovid).

	Omicron variant N=21,231	Delta variant N=21,231	P-value
	N (%)	N (%)	
Outcome-disease status			$2.2x10^{-16}$
Mild	19,391 (91.3)	18,173 (85.6)	
Moderate	1,102 (5.2)	1,229 (5.8)	
Severe/critical	738 (3.5)	1,829 (8.6)	
Moderate or severe/critical outcome	1,840 (8.7)	3,058 (14.4)	

Moderate disease: Hospitalized but no intensive care unit admission; Severe/critical disease: intensive care unit admission or death within 28 days of positive test.

Comparisons between groups were performed using Pearson's X^2 test. A two-sided P-value of < 0.05 was considered to be statistically significant. Adjustments for multiple comparisons were not made.

Supplementary Table 6. Overview of self-reported racial categories included in matched cohort analyses

	Omicron variant N=22,841	Delta variant N=22,841
	N (%)	N (%)
Self-reported racial category		
White	18,811 (82.4)	18,811 (82.4)
Black or African American	2,638 (11.5)	2,638 (11.5)
Asian	143 (0.6)	110 (0.5)
American Indian or Alaska Native	136 (0.6)	180 (0.8)
Native Hawaiian or Other Pacific Islander	165 (0.7)	126 (0.5)
Missing	948 (4.2)	976 (4.3)
