# Supplementary material for: COVID-19 disease severity in US Veterans infected during Omicron and Delta variant predominant periods
Source: Nat Commun. 2022 Jun 25;13:3647. doi: 10.1038/s41467-022-31402-4 (PMC9233663; doi:10.1038/s41467-022-31402-4)
Supplement: Supplementary file 2 — Reporting Summary [file 41467_2022_31402_MOESM2_ESM.pdf]

nature portfolio

Corresponding author(s): Mayr / Butt

Last updated by author(s): 05-23-2022

Reporting Summary

Nature Portfolio wishes to improve the reproducibility of the work that we publish. This form provides structure for consistency and transparency in reporting. For further information on Nature Portfolio policies, see our Editorial Policies and the Editorial Policy Checklist.

Statistics

For all statistical analyses, confirm that the following items are present in the figure legend, table legend, main text, or Methods section.

n/a	Confirmed	
☐	☒	The exact sample size (*n*) for each experimental group/condition, given as a discrete number and unit of measurement
☐	☒	A statement on whether measurements were taken from distinct samples or whether the same sample was measured repeatedly
☐	☒	The statistical test(s) used AND whether they are one- or two-sided<br>*Only common tests should be described solely by name; describe more complex techniques in the Methods section.*
☐	☒	A description of all covariates tested
☐	☒	A description of any assumptions or corrections, such as tests of normality and adjustment for multiple comparisons
☐	☒	A full description of the statistical parameters including central tendency (e.g. means) or other basic estimates (e.g. regression coefficient) AND variation (e.g. standard deviation) or associated estimates of uncertainty (e.g. confidence intervals)
☐	☒	For null hypothesis testing, the test statistic (e.g. *F*, *t*, *r*) with confidence intervals, effect sizes, degrees of freedom and *P* value noted<br>*Give P values as exact values whenever suitable.*
☒	☐	For Bayesian analysis, information on the choice of priors and Markov chain Monte Carlo settings
☒	☐	For hierarchical and complex designs, identification of the appropriate level for tests and full reporting of outcomes
☒	☐	Estimates of effect sizes (e.g. Cohen's *d*, Pearson's *r*), indicating how they were calculated

Our web collection on statistics for biologists contains articles on many of the points above.

Software and code

Policy information about availability of computer code

Data collection	No software was used for data collection.
Data analysis	The R programming code used for data analysis is available at https://github.com/mayrfb/Omicron_Delta_Severity

For manuscripts utilizing custom algorithms or software that are central to the research but not yet described in published literature, software must be made available to editors and reviewers. We strongly encourage code deposition in a community repository (e.g. GitHub). See the Nature Portfolio guidelines for submitting code & software for further information.

Data

Policy information about availability of data

All manuscripts must include a data availability statement. This statement should provide the following information, where applicable:

- The data for this study was created and collected by the VA COVID-19 Shared Data Resource and resources and facilities of the Department of Veterans Affairs (VA) Informatics and Computing Infrastructure (VINCI). VA data are made freely available to researchers behind the VA firewall with an approved VA study protocol. More information is available at https://www.virec.research.va.gov or the VA Information Resource Center (VIReC) at VIReC@va.gov.

Provide your data availability statement here.

Field-specific reporting

Please select the one below that is the best fit for your research. If you are not sure, read the appropriate sections before making your selection.

☒ Life sciences ☐ Behavioural & social sciences ☐ Ecological, evolutionary & environmental sciences

For a reference copy of the document with all sections, see nature.com/documents/nr-reporting-summary-flat.pdf

Life sciences study design

All studies must disclose on these points even when the disclosure is negative.

Sample size	Our sample size was determined by the number of veterans diagnosed with COVID-19 during the study period. We conservatively estimated that 15% of veterans with Delta infection would experience moderate disease to determine the sample size needed for comparisons of disease severity between delta and omicron periods. We considered a 25% reduction in this outcome to be clinically relevant and estimated that a minimum sample size of 2,544 (1,272 in each group) would detect the difference at an alpha level of 0.05 with a power of 80%.
Data exclusions	We excluded observations that did not meet the predefined study time period (delta and omicron, respectively). We also excluded veterans who did not have at least 2 primary care visits prior to vaccine role out.
Replication	We repeated our analyses 4 times including multiple sensitivity analyses and were able to reproduce our main findings.
Randomization	We matched each person in the Omicron variant period to a person in the Delta variant period using random coarsened exact matching. Individuals were matched on age, sex, race, vaccination status at the time of infection, second vaccine dose administration date, Charlson Comorbidity Index, area deprivation score (as marker of socioeconomic status) and VA medical center, to account for local differences in SARS-CoV-2 transmission, testing, and hospital admission practices.
Blinding	This was an retrospective observational trial, hence blinding was not applicable.

Reporting for specific materials, systems and methods

We require information from authors about some types of materials, experimental systems and methods used in many studies. Here, indicate whether each material, system or method listed is relevant to your study. If you are not sure if a list item applies to your research, read the appropriate section before selecting a response.

Materials & experimental systems

n/a	Involved in the study	
☒	☐	Antibodies
☒	☐	Eukaryotic cell lines
☒	☐	Palaeontology and archaeology
☒	☐	Animals and other organisms
☐	☒	Human research participants
☒	☐	Clinical data
☒	☐	Dual use research of concern

Methods

n/a	Involved in the study	
☒	☐	ChIP-seq
☒	☐	Flow cytometry
☒	☐	MRI-based neuroimaging

Human research participants

Policy information about studies involving human research participants

Population characteristics	Veterans 18 years or older with confirmed SARS-CoV-2 infection between October 1, 2021 and January 15, 2022 were eligible for inclusion. Among those, we retained those with at least two primary-care appointments in the preceding 18 months of vaccine roll-out. We excluded veterans who had evidence of prior SARS-CoV-2 infection and veterans who had received non-mRNA vaccines. Our analysis cohort had a median age of 62 years, was predominantly male (91.9%) and 82.4% were White patients. The majority of included subjects were multi-morbid, and the median Charlson Comorbidity Index was 3 (IQR 2-4).
Recruitment	EHR data of veterans with confirmed COVID-19 diagnosis who were treated within the VA healthcare system. Potential biases include misclassification bias of vaccination status and COVID-19 diagnosis.
Ethics oversight	VA Pittsburgh IRB

Note that full information on the approval of the study protocol must also be provided in the manuscript.
